# TIM-3 regulates the proliferation by BDNF-mediated PI3K/AKT axis in the process of endometriosis

**DOI:** 10.1186/s10020-023-00768-6

**Published:** 2023-12-19

**Authors:** Wei Tian, Min Liu, Yuqiu Liu, Qingfeng Lv, HuaFeng Cheng, Yanling Gu, Mingjiang Li

**Affiliations:** 1 Department of Gynecology, Shandong Provincial Hospital Affiliated to Shandong First Medical University, Jinan, Shandong China; 2 Medical Science and Technology Innovation Center, Shandong First Medical University, Jinan, China; 3 Department of Obstetrics, Shandong Provincial Hospital Affiliated to Shandong First Medical University, Jinan, Shandong China

**Keywords:** T cell immunoglobulin and mucin domain-containing molecule-3, Endometriosis, PI3K/AKT, LY294002, Brain-derived neurotrophic factor, Endometrial stromal cells, Proliferation, Transcriptome sequencing technique

## Abstract

**Background:**

T cell immunoglobulin and mucin domain-containing molecule-3 (TIM-3) initially discovered on the surface of Th1 cells, negatively regulates immune responses and mediates apoptosis of Th1 cells. An increasing number of studies have since shown that TIM-3 is crucial in the genesis and development of immune diseases, cancers, and chronic infectious illnesses. However, the effect of TIM-3 on endometriosis is still unknown.

**Methods:**

Quantitative real-time polymerase chain reaction, western blotting, and immunohistochemistry were used to measure TIM-3 levels in endometriosis. Cell Counting Kit-8, 5-ethynyl-2’-deoxyuridine, colony-forming, Transwell® migration, Matrigel® invasion, and flow cytometry assays were used to explore the function of TIM-3 in vitro, and xenograft experiments in nude mice were used to assess its role in vivo. According to the RNA seq, brain-derived neurotrophic factor (BDNF) was screened. The involvement of specific proliferation-related signaling molecules was determined by transfecting a plasmid and adding an inhibitor in vivo and in vitro.

**Results:**

TIM-3 mRNA and protein expression levels were significantly higher in eutopic and ectopic endometrial tissues than in normal endometrial tissues. By examining the effects of TIM-3 overexpression and knockdown on cell proliferation, migration, and invasion in vitro, and lesions formation in vivo, we found that the expression of TIM-3 was positively correlated with cell proliferation and clone formation in vitro, as well as lesions growth in nude mice. By adding the phosphatidylinositol 3 kinase/protein kinase B(PI3K/AKT) pathway inhibitor LY294002 and knocking down PI3K, we further verified that TIM-3 promotes proliferation in vivo and in vitro via the PI3K pathway. By transfecting the plasmid into ESC cells and gave inhibitors to endometriotic rats models, we tested that TIM-3 regulates the proliferation by BDNF-mediated PI3K/AKT axis.

**Conclusion:**

TIM-3 can promote the proliferation of endometriosis by BDNF-mediated PI3K/AKT axis in vivo and in vitro, which may provide a new therapeutic target for the treatment of endometriosis.

**Supplementary Information:**

The online version contains supplementary material available at 10.1186/s10020-023-00768-6.

## Introduction

Endometriosis (EMs) is a common disease in women of childbearing age. Endometriosis has long been one of the leading causes of infertility (Ozkan et al. 2008), with an incidence of up to 50% in infertile patients. Furthermore, dysmenorrhea, lower abdominal pain, dyspareunia, and other symptoms caused by the disease seriously affect the physical and mental health of women with endometriosis. Endometriosis is characterized by active endometrial cells that take hold outside the endometrium, causing ectopic growth, infiltration, and repeated bleeding. Although endometriosis is a benign disease, it has features in common with malignant tumors, such as abnormal proliferation, metastasis, invasion, etc., and is hence sometimes referred to as a ‘benign cancer’. Patients with endometriosis often exhibit local and systemic immune dysfunctions. Although many correlational studies have been conducted on endometriosis, such as retrograde menstruation, chronic inflammation, and aberrant angiogenesis (Laganà et al. 2019), the exact etiology and mechanism of endometriosis remain unclear. Currently, the most globally shared etiological theory is menstrual blood reflux, which means that menses enter the pelvic cavity through the oviduct, take the endometrium that falls off during the menstrual period into the pelvic cavity, and become implanted in the uterine rectal fossa, ovary, or other positions. However, approximately 90% of women have menstrual reflux, but only 10% exhibit endometriosis (Tang et al. 2021), suggesting that menstrual reflux is one of several causes of endometriosis. A number of studies have shown changes in the expression of genes related to proliferation, migration, and apoptosis, leading to a change in the function of eutopic or ectopic endometrial cells, thus providing the basic conditions for ectopic implantation (Li et al. 2014; Meng et al. 2019). Therefore, further research is needed on the mechanisms of endometriosis to explore new treatments.

The T cell immunoglobulin and mucin-domain protein family (TIM) has been implicated in allergic reactions and has homologous chromosomal regions in humans (5q33.2) and mice (11B1.1) (McIntire et al. 2001). TIM-3, originally called hepatitis A virus cellular receptor 2 (HAVCR2), was first reported to be a cell surface immunomodulator in 2002. It is encoded by the HAVCR2 gene, which is expressed extensively in CD4 + Th1 cells, which secrete IFN-γ, CD8 + T cytotoxic (Tc1) cells, Th17 cells, regulatory T cells (Treg), dendritic cells (DCS), natural killer cells (NK), and monocytes (Monney et al. 2002) The membrane segment of TIM-3 is a single transmembrane domain. The overall structure of TIM-3, from the N-terminus to the C-terminus of the peptide chain, comprises an IgV subunit, a mucin-like domain, a single transmembrane domain, and a variable-length Tyr-rich intracellular segment (Lee et al. 2011a, b). The IgV subunit is located on the outside of the cell and, together with the mucin-like domain, participates in ligand recognition. Selective expression of TIM-3 in tumors participates in immunosuppression, indicating its value in cancer immunotherapy. TIM-3 is tightly associated with advanced tumor node metastasis in different tumors, including gastric (Jiang et al. 2013), colon (Zhou et al. 2015), and cervical cancers (Cao et al. 2013). Preclinical studies in a variety of tumor models have demonstrated that blocking TIM-3 limits tumor progression, particularly in combination with PD-1/PD-L1 inhibition (Seo et al. 2018). TIM-3 is preferentially expressed on leukemic progenitor cells, and it has been linked to the immune dysfunction of lymphoid malignant tumors (Jan et al. 2011) (Huang et al. 2010). Overexpression of TIM-3 in renal cell carcinoma CD20 + TAMs and renal cell carcinoma was positively correlated with short progression-free survival (PFS) (Komohara et al. 2015). However, aside from immune-related effects, it is unclear whether TIM-3 has some mechanisms of action in endometriosis. The internal expression of TIM-3 and the effects that TIM-3 has on the development of endometriosis have not been elucidated.

The study of TIM-3 in endometriosis to date has been limited to the immune response in the peritoneal effusion and blood. However, the expression of TIM-3 in endometrial stromal cells (ESCs) and its effects on proliferation migration, and invasion are unknown, and the precise role in the development of EMs has not been explored. In light of its effects on tumors, a crucial assumption is that TIM-3 participates directly in the proliferation of endometriosis cells, which is related to the development of endometriosis. To verify this assumption, we tested the expression of TIM-3 in endometriotic tissues and conducted in vivo and in vitro experiments to demonstrate the influence of TIM-3 on the biological characteristics of endometriosis and its potential mechanisms.

## Materials and methods

### Ethical approval

The research project was approved by the Ethics Committee of the Provincial Hospital of Shandong First Medical University. All patients enrolled in the trial signed an informed consent form to approve use of the samples in the experimental study.

### Patients and tissue samples

The endometriosis group (n = 30) included patients with ovarian ‘chocolate cysts’, ectopic endometrium (Ec) tissues, and eutopic endometrial (Eu) tissues. The control group (n = 30) underwent hysteroscopic endometrial curettage due to simple tubal infertility, thereby providing normal endometrium (NE) tissues. All the patients enrolled in the trial had regular menstrual periods and in the secretory phase, who had not taken any hormone medicines for at least half a year before harvesting. Patients with uterine leiomyoma, chronic pelvic inflammatory disease, endometrial lesions, gynecological cancers, other ovarian cysts, or polycystic ovary syndrome were excluded.

After being harvested from the patients, a part of the specimens was fixed in 4% paraformaldehyde, and a part was placed in liquid nitrogen for immediate preservation. The rest of the eutopic endometrial specimens were stored in phosphate-buffered saline (PBS) and placed in an ice box for primary cell extraction.

### Immunohistochemistry (IHC)

The specimens fixed in 4% paraformaldehyde were embedded in paraffin and sliced into sections with a thickness of 4 μm. The paraffin-embedded sections were then placed in an oven at 60℃ for one hour to evaporate and melt the paraffin, followed by dewaxing with xylene and hydration with a gradient of alcohol. We used a pH = 6.0 citric acid buffer in a microwave oven for antigen thermal repair and 3% hydrogen peroxide for 15 min to inactivate the endogenous peroxidase avidity. After being blocked with normal sheep serum at ambient temperature for 30 min, the sections were incubated with rabbit monoclonal anti-human TIM-3 (1:300) (ab241332, Abcam, Cambridge, MA, USA) at 4 °C overnight. The next day, the sections were incubated with horseradish peroxidase-labeled goat anti-rabbit IgG (GeneTech, Shanghai, China) at room temperature for 30 min and then stained by DAB (GeneTech) for five minutes. After hematoxylin staining of the nuclei, the sections were dehydrated and mounted.

Two experienced pathologists scored the slides according to the staining intensity and the percentage of positive cells in ten fields of view imaged with a microscope in each case. The scores for no positive staining (negative, -), light yellow (weakly reactive, +), brown (positive, ++), and dark brown (strongly positive, +++) were 0, 1, 2, and 3, respectively. According to the percentage of positive cells, it was rated as four grades, with ≤ 25% as one point, 26–50% as two points, 51–75% as three points, and > 75% as four points. The final points value was obtained by multiplying the two scores, used as the TIM-3 expression (total score zero, negative; total score one to four, weak positive; total score five to eight, positive; total score nine to twelve, strong positive).

### Isolation, culture, and authentication of endometrial stromal cells

Endometrial tissues harvested under sterile conditions were cleaned by washing with PBS and then homogenized. After centrifugation, three volumes of type I collagenase (Sigma-Aldrich, St. Louis, MO, USA) were added and the sample was digested in a 37℃-water bath for forty minutes with vortexing every ten minutes. The digestion was then terminated by the addition of three volumes of DMEM/F-12 medium (DMEM/F-12; Gibco, Grand Island, NY, USA) containing 10% fetal bovine serum (FBS; Ex-Cell Bio, origin: Uruguay) to the sterile samples. The suspensions were then filtered through cell sieves of 154 mm and 40 mm. The cells obtained were centrifuged, resuspended in DMEM/F-12 medium containing 10% FBS, and then cultured in 5% CO2 at 37℃ (MCO-15AC incubator, Sanyo, Japan).

Human endometrial stromal cells were identified using immunofluorescence. The primary ESCs (1 × 10^5^/mL) were seeded on coverslips in 24-well plates and incubated for 24 h. The coverslips were then washed three times with PBS and placed in 4% paraformaldehyde for 30 min. The cells were permeabilized in PBS containing 0.5% TritonX-100 at room temperature for 20 min, blocked with 0.5% bovine serum albumin (BSA) for 30 min, and then incubated with rabbit monoclonal antibodies against vimentin (1:100) (ab16700, Abcam, Cambridge, MA, USA) and E-cadherin (1:200) (ab 231,303, Abcam, Cambridge, MA, USA) at 4℃ overnight. The next day, the coverslips were washed and incubated with fluorescent secondary antibody (1:200) (ab193595, Abcam, Cambridge, MA, USA) for one hour in a black wet box, and DAPI was added to stain the nuclei for ten minutes while avoiding exposure to light. The slides were mounted with gum-sealed tablets containing a fluorescence quenching inhibitor and then imaged using a fluorescence microscope. The human endometrial stromal cells isolated in our study were > 95% pure.

### Cell lines and culture

The human endometrial stromal cell line (hESC), provided by the Qilu Hospital Laboratory of Shandong University, was cultured in MEM-alpha basic medium (Gibco) with 10% FBS by incubation in 5% CO_2_ at 37℃.

### Lentivirus transfection and establishment of stable cell lines

An overexpressing lentivirus and three short hairpin RNAs (shRNAs) targeting TIM-3 were generated by Shanghai Genechem Co., Ltd. (Shanghai, China). Transfection of cells with virus was performed according to the manufacturer’s specifications, and the multiplicity of infection (MOI) was 20. Puromycin (5 µg/mL) (Genechem Co, Shanghai, China) was used to select stably transfected cells until the transfection efficiency exceeded 90%. The transfection rate was determined using qRT-PCR and WB.

The TIM-3 overexpression plasmid was constructed by Guangzhou RiboBio Co., Ltd. (Guangzhou, China), combined with Lipofectamine™ 2000 (Zeman Bio Beijing China) and Opti-MEM™ medium (Gibco) to transfect cells. RiboFECT™ CP transfection (Guangzhou RiboBio Co., Ltd. Guangzhou, China) was used to transfect the cells with three different siRNAs targeting TIM-3 and a negative control synthesized by RiboBio Co., Ltd.

### Quantitative real‑time polymerase chain reaction(qRT-PCR)

TRIzol® reagent (Accurate Bio-Medical Technology Co., Ltd., Hunan, China) was added to lyse the cells and tissue homogenates after maceration, and chloroform and isopropanol were added successively according to the manufacturer’s instructions. After using RNase-free water to dissolve the dried RNA precipitate, the purity of the total RNA was determined using a NanoDrop™ device (Thermo Fisher Scientific, Wilmington, DE, USA), and the OD_260/280_ value was 1.8–2.0, indicating that the purity was very high. Using RNA as a template, cDNA was generated using an Evo M-MLV Reverse Transcription Kit including gDNA removal reagent (Accurate Bio-Medical Technology), according to the manufacturer’s instructions. A QuantStudio™ 5 Real-Time PCR System (Thermo Fisher Scientific) and a SYBR® Green Pro Taq HS premixed qPCR kit (Accurate Bio-Medical Technology) were used to perform the qRT‑PCR, which consisted of three basic reaction steps: denaturation, annealing, and extension. The specific settings were as follows: initial denaturation at 95℃ for 30 s, followed by 40 cycles of amplification at 95℃ for 5 s, and 60℃ for 30 s. The gene-specific primers synthesized by AG (Accurate Bio-Medical Technology) were as follows: TIM-3: forward, 5′-TCATCAAACCAGCCAAGGTCA-3′ and reverse, 5′-GTCCCCTGGTAAGCATC-3′; Actin: forward, 5′-GGTTGT CTC CTG CGA CTT CA -3′ and reverse, 5′-TGG TCCAGG GTT TCT TAC TCC-3′, PI3Kp85: forward, 5′-ATGGCA CCT TCC TAG TCC GAGA-3′ and reverse, 5′-CTCTGA GAA GCC ATA GTG CCCA-3′. The 2-^∆∆Ct^ method and Prism software were used to calculate and draw the curves. The experiment was performed independently at least three times.

### Western blotting (WB)

After being washed three times with PBS (1X), tissues or cells were dissociated using Radioimmunoprecipitation (RIPA) Lysis Buffer (Beyotime Biotechnology Shanghai, China) supplemented with protease inhibitor cocktail for 30 min and vortexed every ten minutes. The extracted proteins were centrifuged at 12,000 ×g at 4℃ for 30 min, and the protein concentration was determined using a bicinchoninic acid (BCA) protein quantitative kit (Beyotime) according to the manufacturer’s protocol. The proteins were separated by 10% sodium dodecyl sulfate-polyacrylamide gel electrophoresis (SDS-PAGE) and then electrophoretic ally transferred onto PVDF membranes (Millipore, Billerica, MA, USA). After being washed with TBST, the membranes were blocked with TBST containing 5% BSA for one hour to reduce non-specific adsorption and then incubated with primary antibodies at 4 °C overnight. The next day, the membranes were washed and incubated with horseradish peroxidase (HRP)-conjugated secondary antibodies for 1 h at room temperature. Chemiluminescence reagents (Millipore) were used to detect protein bands with a Fusion FX5 Spectra imaging system. Anti-β-actin, β-tubulin, signals were used as loading controls, and each experiment was performed independently at least three times. Antibodies: TIM-3 (ab241332, 1:1000, Abcam, Cambridge, USA), PI3K (T40064, 1:1000, ab-mart, Shanghai, China), pPI3K (TA32421:1000, ab-mart, Shanghai, China), AKT (T55561, 1:1000, ab-mart, Shanghai, China), pAKT (T40067, 1:1000, ab-mart, Shanghai, China), BDNF (GB11559,1:1000, Servicebio, Wuhan, China), TrkB (GB11295-1, 1:1000, Servicebio, Wuhan, China), β-actin(P30002, 1:2000, ab-mart, Shanghai, China), β-tubulin(M30109, 1:2000, ab-mart, Shanghai, China), Goat Anti-Rabbit Mouse IgG-HRP(M21003, 1:5000, ab-mart, Shanghai, China).

### Cell proliferation bioassay

Cell Counting Kit-8 (CCK8) and 5-ethynyl-2’-deoxyuridine (EDU) assays were used to measure the proliferation of ESC. The transfected ESCs in their second generation were cultured in 96-well plates for 24, 48, and 72 h, and 10 µL of CCK8 reagent (Solarbio Life Sciences, Beijing, China) was added at the same time every day and incubated for one hour. A Multiskan Sky High full-wavelength microplate reader (Thermo Scientific™) was used to determine the optical density (OD) at 450 nm.

Six-well plates were used to culture and transfect with siRNAs and plasmid for 24 h. The transfected cells were then reseeded at 3000 cells/well in 6-well plates and cultured for 24 h. An Edu Cell Proliferation Kit with Alexa Fluor 555 (Epizyme Biomedical Technology Co., Ltd., Shanghai, China) and an inverted fluorescence microscope (Olympus Corporation, Tokyo, Japan) were used.

### Colony-formation assay

TIM-3 overexpression/knockdown hESCs were seeded into 6-well plates at 1000 cells per well and incubated for two weeks or until the number of cells for most single clones was more than 50, which required changing the medium every three days and observing the cell status. The cells were then placed in methanol for 30 min and stained with 1% hexamethyl pararosaniline for 20 min. Colonies containing > 50 cells were counted. The formula for calculating the colony formation rate was as follows: cloning efficiency = (colony formation number/cultured cell number) ×100%.

### Wound-healing assay

Before the experiment, horizontal lines were drawn on the 6-well plate (with a ruler), with at least five lines passing through each well, and each line was uniform and parallel. Stably transfected ESCs were then inoculated at a density of 3 × 10^6^ cells per well. The next day, when the cells reached more than 80% confluence, a scratch was made in the cell monolayer with a 200 µL pipette tip. The wells were washed three times with PBS (1X) to remove the unattached cells and to make the monolayer gap visible to the naked eye, and the liquid was then replaced with fresh serum-free medium. Images were taken at 0, 12, and 24 h using an EVOS™ XL Core microscope (Olympus). At each time point, five random fields were used to compare the widths of the original scratched areas.

### Transwell® migration assay

After adding the Transwell® chamber to the 24-well culture plate, the chamber was designated as the superior chamber, and the culture plate was designated as the inferior chamber. The pore diameter of the Transwell® filter membrane (Corning, Kennebunk, ME, USA) was 8 μm. TIM-3-overexpression/knockdown transfected ESCs were trypsinized and suspended in DMEM/F-12 basic medium without FBS. The cell suspension was placed into the superior chamber according to 200 µL (1 × 10^5^ cells) per well, and 500 µL DMEM/F-12 basic medium containing 20% FBS was placed into the inferior chamber, which was cultured at 37℃ for twenty-four hours. Subsequently, the chambers were fixed with methanol for 30 min, dyed with 0.5% hexamethyl pararosaniline for ten minutes, rinsed slowly with running water, and the water was gently absorbed by a cotton swab in the upper room at the end. Each chamber was randomly counted in five fields using a microscope and photographed.

### Matrigel® transwell® invasion assay

Corning Matrigel® (Corning, Kennebunk, ME, USA) was removed from the minus 20 °C freezer and placed at 4 °C one day in advance. After dilution with DMEM/F-12 basic medium without FBS at 1:7, Matrigel® (100 µL) was applied to each upper chamber to avoid bubbles and placed at 37℃ for 1 h to solidify. The cell suspension was placed into the superior chamber at 200 µL (5 × 10^5^ cells) per well, and the remaining steps were the same as in the Transwell® migration experiment. Five random fields in each chamber were imaged using a microscope.

### Flow cytometry apoptosis assay

Annexin V-fluorescein isothiocyanate (FITC)/Propidium Iodide (PI) staining assay kits (Meilunbio Biotechnology Co., Ltd., Dalian, China) were used to evaluate ESC apoptosis by flow cytometry, following the manufacturer’s instructions. After digestion with trypsin without EDTA, the cells were collected by centrifugation at 800×g for five minutes, washed twice with PBS, and resuspended in 1x Binding Buffer to obtain a cell concentration of 1 × 10^6^ cells/mL. FITC and PI were added while avoiding light exposure. Normal cells, PI-stained cells, and FITC-stained cells were used as control groups. An acoustic focusing flow cytometer (Thermo Fisher Scientific) was used to detect FITC and PI fluorescence.

### Xenograft growth in nude mice

Female BALB/c nude mice, aged four weeks, were purchased from Beijing Charles River Laboratory Animal Technology Co. (Beijing, China). The ESCs was isolated from nine patients and divided into three groups to transfect lentivirus targeting TIM-3. In the first experiment, the mice were divided into three groups, with five mice in each group: a TIM-3 NC group, a TIM-3 shRNA group, and a TIM-3 OE group, which were raised in a sterile purification barrier system with an indoor temperature of 26–28 °C, relative humidity of 40–60%, and a high-efficiency filter for ventilated airflow. One week after the nude mice were introduced into the Animal Research Center of Shandong Provincial Hospital, each nude mouse was injected under the right forelimb with 200 mL (1.0 × 10^7^/mL) of cells resuspended in PBS. After delivery of the cell suspension, the syringe was slowly pulled out and a cotton swab was applied for a short time. The earliest ectopic foci were observed on the fourth day after injection, and all mice exhibited foci on the seventh day. The longest (A) and shortest (B) diameters of the ectopic foci in nude mice were measured and recorded with Vernier calipers every three days and used to calculate the volume using the formula: 0.5× (AxB^2^). Mice were killed on the twenty-sixth day after injection, the ectopic foci were removed, and the weights were recorded. Part of the foci was fixed with paraformaldehyde to prepare wax blocks, and part of the tissue was immediately placed in an ultra-low temperature freezer for subsequent experiments.

In the second experiment, the mice were divided into three groups, with six mice in each group: a Tim-3 NC group, a TIM-3 OE + LY294002 group, a TIM-3 OE group, which were raised in the same system as the first batch. One week after the injection, LY294002 (50 mg/kg) was administered intraperitoneally every four days, and the mice were killed on the 25th days after modeling.

### Transcriptome sequencing technique (RNA-seq)

RNA preparation, library construction, and sequencing were performed by Genechem using Illumina sequencing platform. Total RNA was generated from ESCs after affected according to the protocol. The samples were homogenized in TRIzol® reagent. In order to determine whether the sequencing data were suitable for subsequent analysis, raw RNA sequencing data required quality control (QC) on the raw reads. The resulting clean data were aligned to the reference sequence and low-quality reads were discarded after QC.

### An endometriosis model in rats

Female Sprague-Dawley (SD) rats (n = 16; weight ~ 200 g;7weeks) were purchased from Beijing Vital River Laboratory Animal Technology Co., Ltd. and housed in a specific pathogen‑free environment that was automatically maintained at a temperature of 23 ± 2˚C, a relative humidity of 45‑65%, and with a controlled 12 h light/dark cycle. The animals had free to access food and water. The estrous cycles of rats were confirmed by vaginal smears. The rats in estrus were anesthetized by Aver din and fixed on the operating table. Iodophor was uniformly smeared over the abdomen, which was then cut open to locate the uterus. Two sides of the uterus were ligated, along with the vessels, by twine and 1 cm was left in the middle. This 1 cm section of tissue was cut using scissors and placed in a sterile culture dish containing normal saline. The endometrium was removed using sterile microscopic tweezers and cut into fragments measuring 5*5 mm. These fragments were attached to the abdominal wall. The incision was stitched and smeared with iodophor.

Following the induction of endometriosis, the rats were allowed to recover for 4 weeks, during which time they were not administered with any medication. Subsequently, 16 rats were divided into the following four experimental groups: Control (PBS), Tim-3 inhibitor (BP0115, Bio X Cell, LLC, 3 mg/kg, intravenous), K252a (HY-N6732, Med Chem Express, 20 mg/kg, intravenous), Tim-3 inhibitor + K252a(intravenous). The drugs were given every three days and lasted for three weeks, then the rats were killed by carbon dioxide after collecting specimens including hearts, livers, spleens, lungs, kidneys, and foci. Body mass was recorded every 3 days in the period of the experiment.

### Statistics analysis

Prism 7.0 (GraphPad, USA) and SPSS software (version 25.0; SPSS Inc., Chicago, IL, USA) were used for the statistical analyses. Student’s t-test and one-way ANOVA were used to compare the data of the two groups. All data are reported as means ± the SD. *p* < 0.05 was considered statistically significant.

## Results

### Expression of TIM-3 in endometriosis

**Fig. 1:**
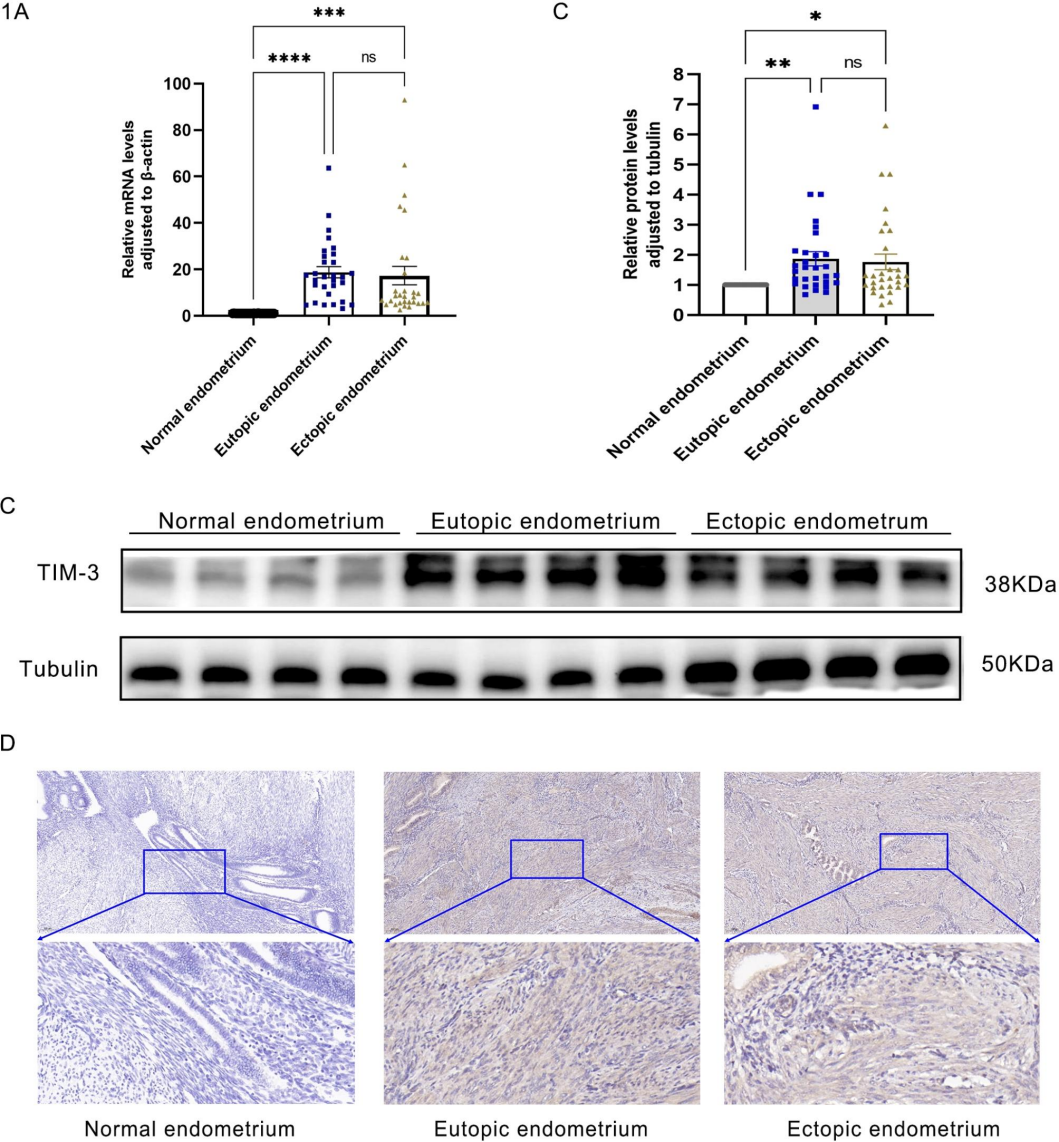
TM-3 expression is upregulated in the eutopic and ectopic endometrium of endometriosis patients compared with the control endometrium. (**A**) T-cell immunoglobulin and mucin domain-containing molecule-3 (TIM-3) mRNA levels in the eutopic and ectopic endometrium of endometriosis patients were upper than those in the control endometrium, as detected by quantitative RT-PCR (qRT-PCR) (* P < 0.05). Data were normalized to β-actin. Results are mean ± SD of three independent experiments. Data were analyzed by Student’s t-test. (**B**) TIM-3 protein levels in the eutopic and ectopic endometrium of endometriosis were upper than those in the control endometrium, detected by western blot. β-tubulin was an internal control. (**C**)TIM-3 protein was ubiquitously expressed on the plasma membrane and in the cytoplasm of Eu and Ec. (*p < 0.05, **p < 0.01, ***p < 0.001, ****p < 0.0001)

The qRT-PCR and WB results of 30 patients in each group showed that TIM-3 expression in Eu and Ec was higher than that in NE, but there was no statistical significance between Eu and Ec (Fig. 1A and B). To further verify the above results, IHC was used, and the results of 30 patients in each group showed that TIM-3 protein was ubiquitously expressed on the plasma membrane and in the cytoplasm of Eu and Ec. (Fig. 1C). In addition, we conducted an assessment of vascular endothelial growth factor (VEGF) and estrogen receptor-β (ER-β) expression as a complementary measure to validate the precision of our sample collection process (Fig S1).

### Validation of the transfection rate and endometrial stromal cells

**Fig. 2:**
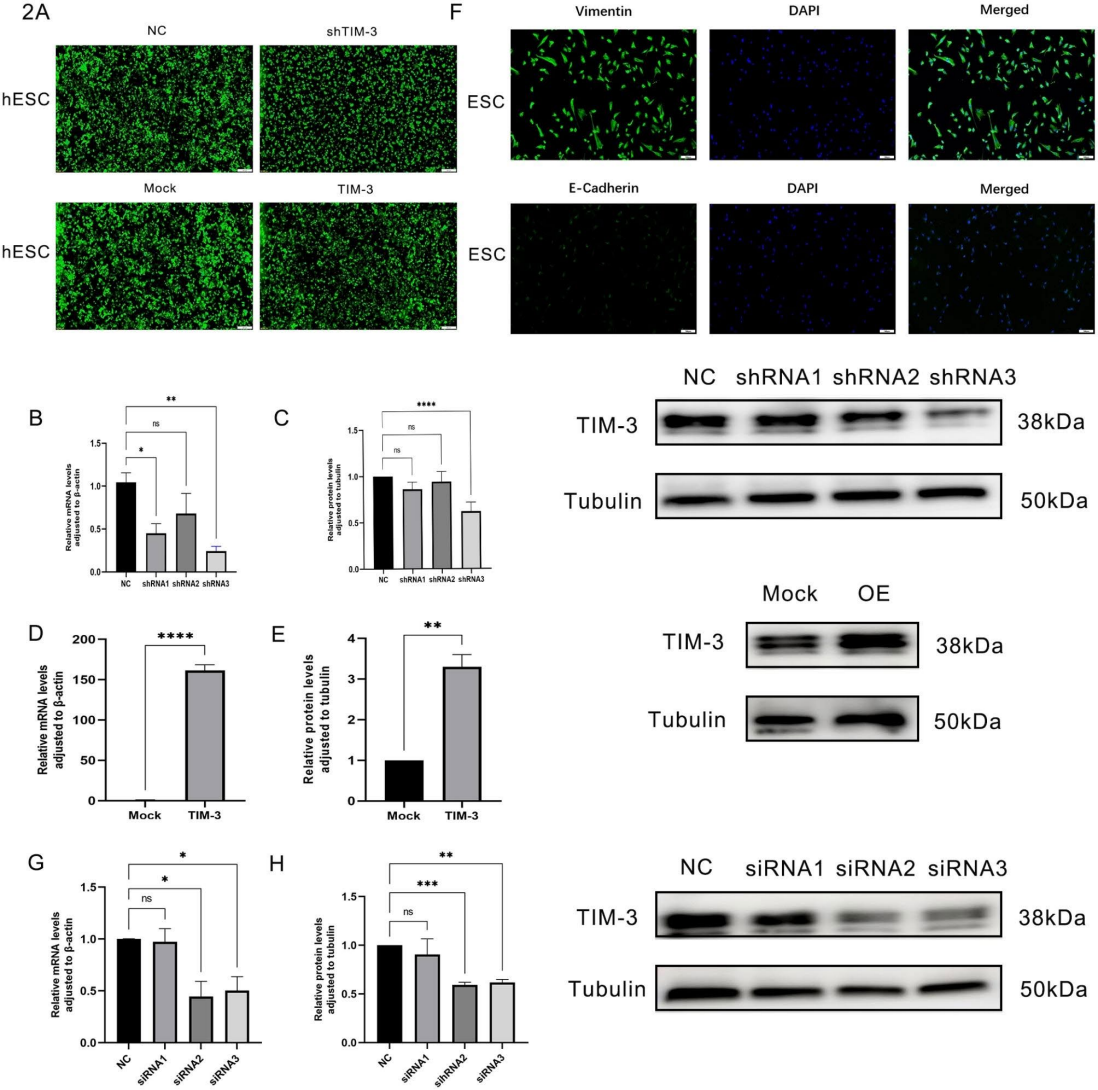
Knockdown and Overexpression of TIM-3. (**A**). fluorescence detected the transfection of cell lines, Magnification,100 ×. (**B**, **C**) Downregulation of TIM-3 was assessed by qRT-PCR and western blot analysis after transfection with three shRNAs or the negative control (NC). The third shRNA was selected for further investigation. (**D**, **E**). Upregulation of TIM-3 assessed by qRT-PCR and western blot analysis. (**F**) fluorescence detection of a Primary culture and identification of ESCs (strongly positive of vimentin > 95%, negative of E-cadherin). Magnification,100 ×. (**G**, **I**) Downregulation of TIM-3 was assessed by qRT-PCR and western blot analysis after transfection with three siRNAs or the negative control (NC). The second siRNA was selected for further investigation. (*p < 0.05, **p < 0.01, ***p < 0.001, ****p < 0.0001)

To clarify the effect of TIM-3 knockdown on hESC, we transfected hESCs with three lentiviral interfering vectors targeting TIM-3. After transfection of the cell lines, fluorescence detection was performed (Fig. 2A). The transfection efficiency was assessed by qRT-PCR and WB, which showed that the knockdown of TIM-3 expression by shRNA3 was effective (Fig. 2B and C). To clarify the role of TIM-3 overexpression in hESC cells, we transfected cells with a lentivirus overexpressing TIM-3, and the overexpression efficiency of TIM-3 was analyzed at the mRNA and protein levels, which revealed that the overexpression of TIM-3 by the lentivirus was successful in hESC cells (Fig. 2D and E).

Staining for the mesenchymal-specific marker vimentin was positive, whereas the staining for the epithelial marker E-cadherin was negative. To verify this result, primary endometrial stromal cells were successfully isolated (> 95%) (Fig. 2F). Three small interfering RNA (siRNA) targeting TIM-3 were constructed, and the interference efficiency was verified by qRT-PCR and WB. The results showed that the knockdown of TIM-3 expression by siRNA2 was effective (Fig. 2G and I).

### TIM-3 enhances proliferation, colony-formation, and inhibits ESCs apoptosis

**Fig. 3:**
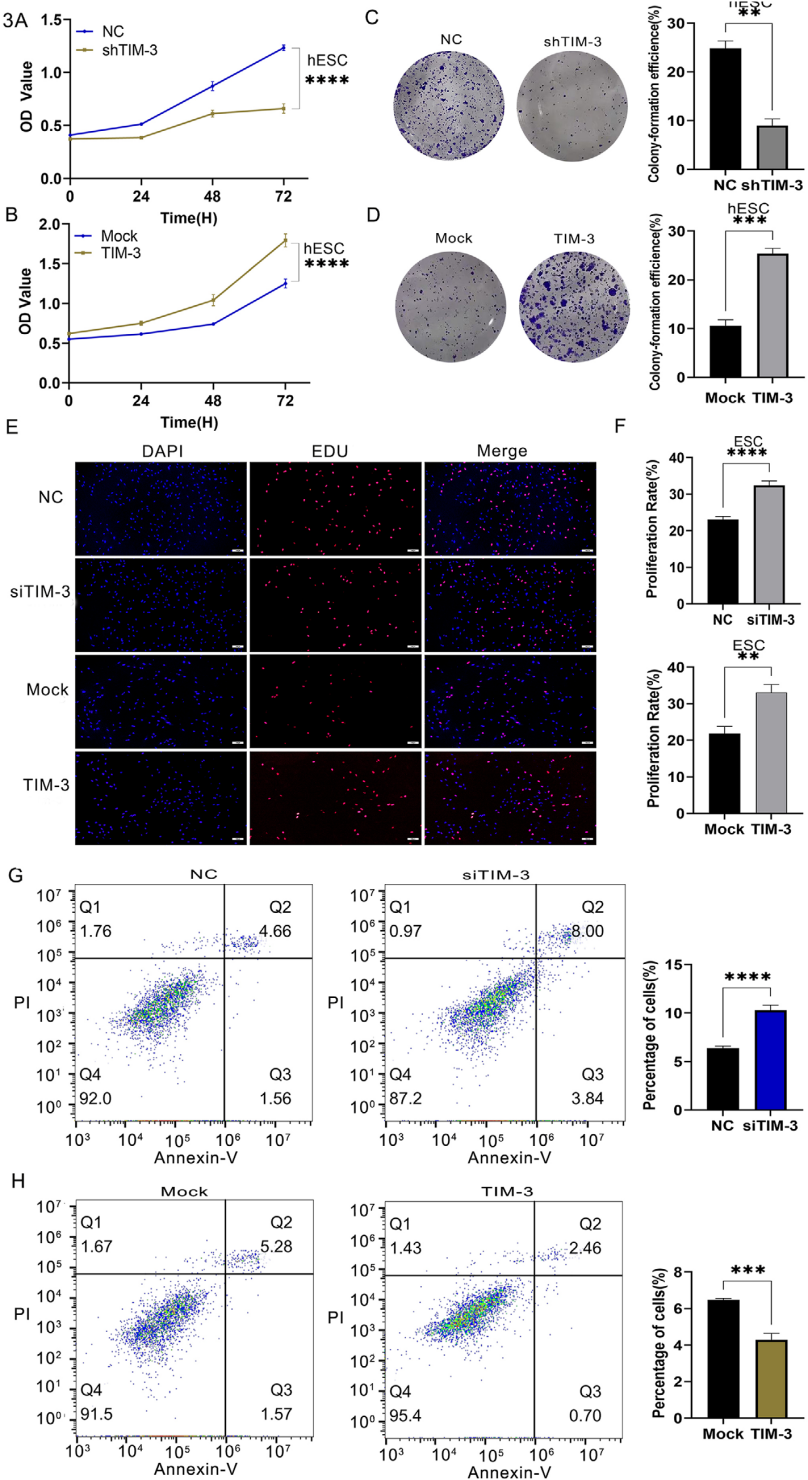
Effects of TIM-3 downregulation and overexpression on cell proliferation and apoptosis. (**A**, **B**) Downregulation of TIM-3 inhibited cell proliferation of hESCs, detected by CCK8 and clone-forming assay, and overexpression did the opposite effect. (**C**, **D**) TIM-3 affected in the clone-forming ability of hESCs. (**E**, **F**) Downregulation of TIM-3 inhibited cell proliferation of ESCs, detected by EDU assay. Overexpression of TIM-3 promoted cell proliferation of ESCs. (**G**, **H**) TIM-3 knockdown promoted the apoptosis, and TIM-3 overexpression inhibited ESCs apoptosis. (*p < 0.05, **p < 0.01, ***p < 0.001, ****p < 0.0001)

To determine the effect of TIM-3 on the growth of endometrial cells, the hESCs were transfected and then evaluated by CCK8 and colony-forming assays. After ESCs were isolated and transfected, EDU and apoptosis detection were used to verify the effects. The optical density (OD) of the control group was greater than that of the TIM-3 shRNA group and lower than that of the TIM-3 OE group (Fig. 3A, B), which was verified by the colony-forming assay (Fig. 3C, D). The EDU assay yielded the same results as the two assays above, confirming that TIM-3 plays a vital role in the proliferation of ESCs (Fig. 3E). TIM-3 knockdown promoted the apoptosis of ESCs (Fig. 3G), while TIM-3 overexpression inhibited ESCs apoptosis (*p* < 0.05) (Fig. 3H).

### TIM-3 overexpression/knockdown affect migration and invasion

**Fig. 4:**
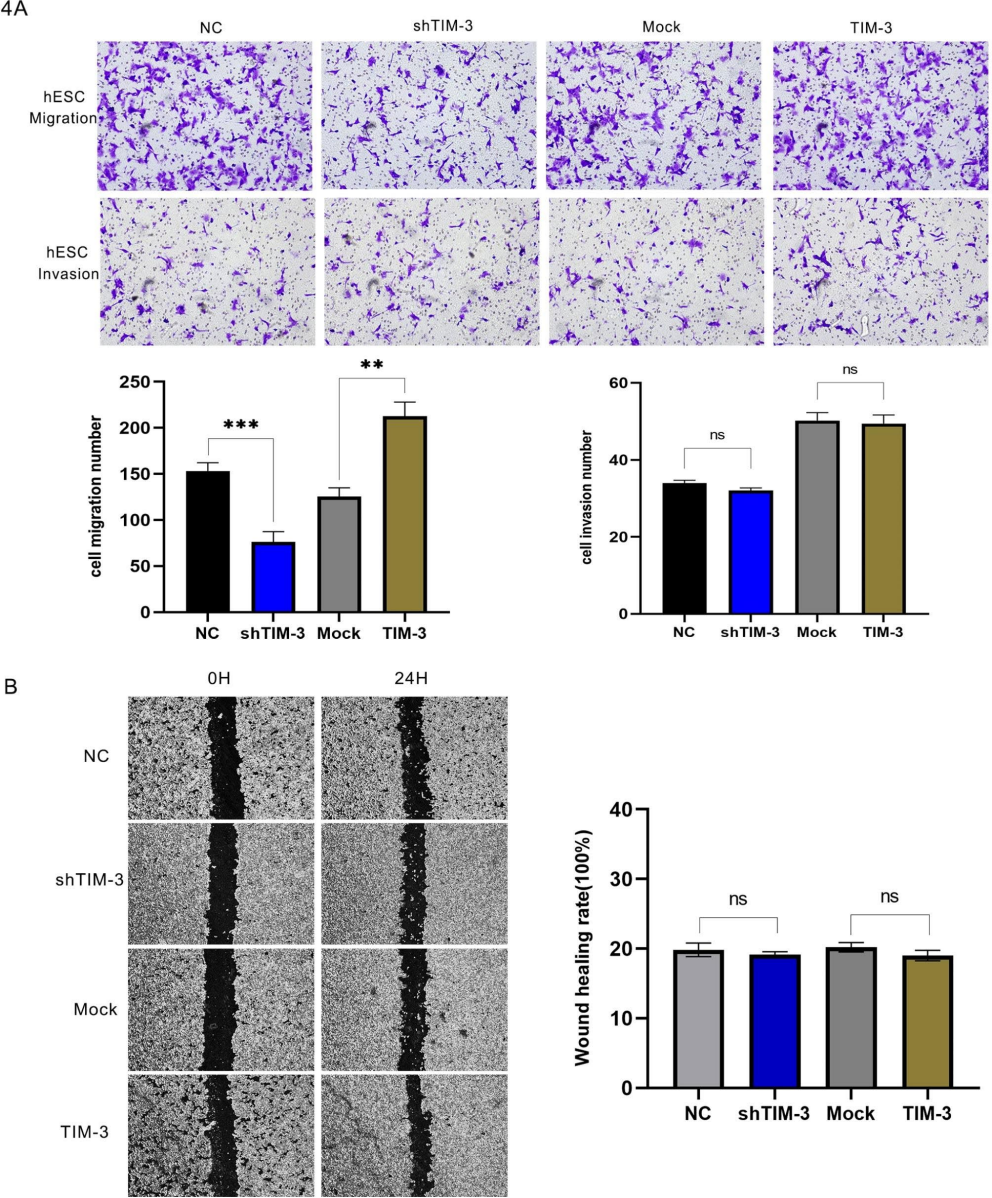
Effects of TIM-3 downregulation and overexpression on cell invasion, migration, and wound healing. (**A**) Down-regulation and overexpression of TIM-3 influenced migration, while they had no effect on the invasion of hESCs, assessed in a Transwell® assay. (**B**) Down-regulation and overexpression of TIM-3 had no effect on the migration of ESCs, assessed in wound healing assay. (*p* > 0.05). (*p < 0.05, **p < 0.01, ***p < 0.001, ****p < 0.0001)

Wound healing, Transwell® migration, and invasion assays were used to assess the effect of TIM-3 on the metastatic capacity. As shown in Fig. 4A, TIM-3 knockdown or overexpression had a significant effect on cell migration measured by Transwell® and migration assays (*p* < 0.05). However, TIM-3 knockdown or overexpression did not affect the invasion ability (*p* > 0.05). The results of the wound healing assay (Fig. 4B) showed that there was not a significant effect on cell migration in the TIM-3 knockdown or overexpression groups (*p* > 0.05).

### TIM-3 promotes endometriotic lesion growth in vivo

**Fig. 5:**
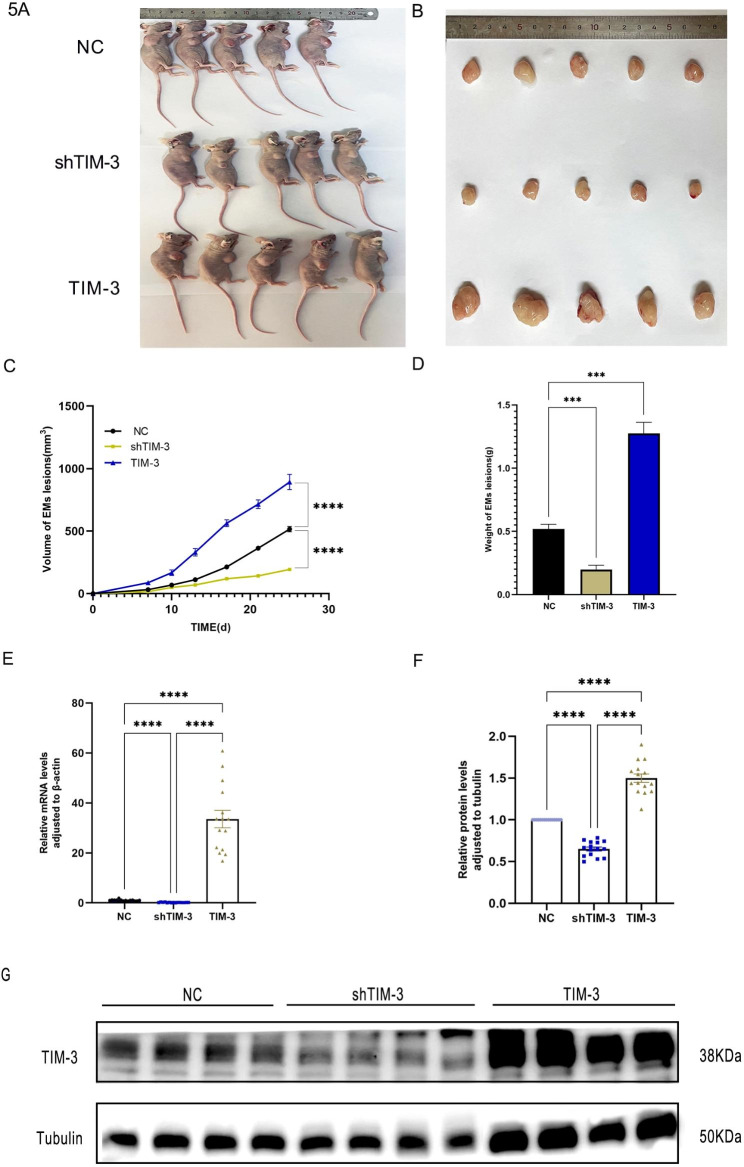
Upregulation of TIM-3 accelerates tumor growth in vivo. Transfected (1.0*10^7^/ml) cells were subcutaneously implanted. (**A**). Images of nude mice (**B**). tumors from all groups (**C**, **D**). the growth curve of tumor size and the tumor weight at the end of the experiments. (**E**) TIM-3 expression assessed by qRT-PCR. (**F**, **G**). TIM-3 expression assessed by western blot Data shown as mean ± SD. (*p < 0.05, **p < 0.01, ***p < 0.001, ****p < 0.0001)

An endometriosis model was established in nude mice to examine whether TIM-3 plays a similar role in the proliferation of endometriosis cells in vivo and in vitro. The armpits of BALB/c nude mice were injected with TIM-3 shRNA, TIM-3 NC (control), or TIM-3 OE cells to generate the endometriosis mouse model (Fig. 5A). Twenty-six days after injection, the mice were sacrificed, and the endometriotic lesions were excised. Visible to the naked eye, the slowest growing group was the TIM-3 shRNA group, whereas the fastest growing group was the TIM-3 OE group (Fig. 5B). Further analysis of the lesion volumes and weights confirmed that the TIM-3 shRNA group had significantly inhibited growth (Fig. 5C, D). The results of qRT-PCR and WB further verified that TIM-3 expression was higher in the TIM-3 OE group than in the TIM-3 shRNA group (Fig. 5E, F, G).

### TIM-3 promotes the proliferation of endometriosis cells through the PI3K/AKT pathway

**Fig. 6:**
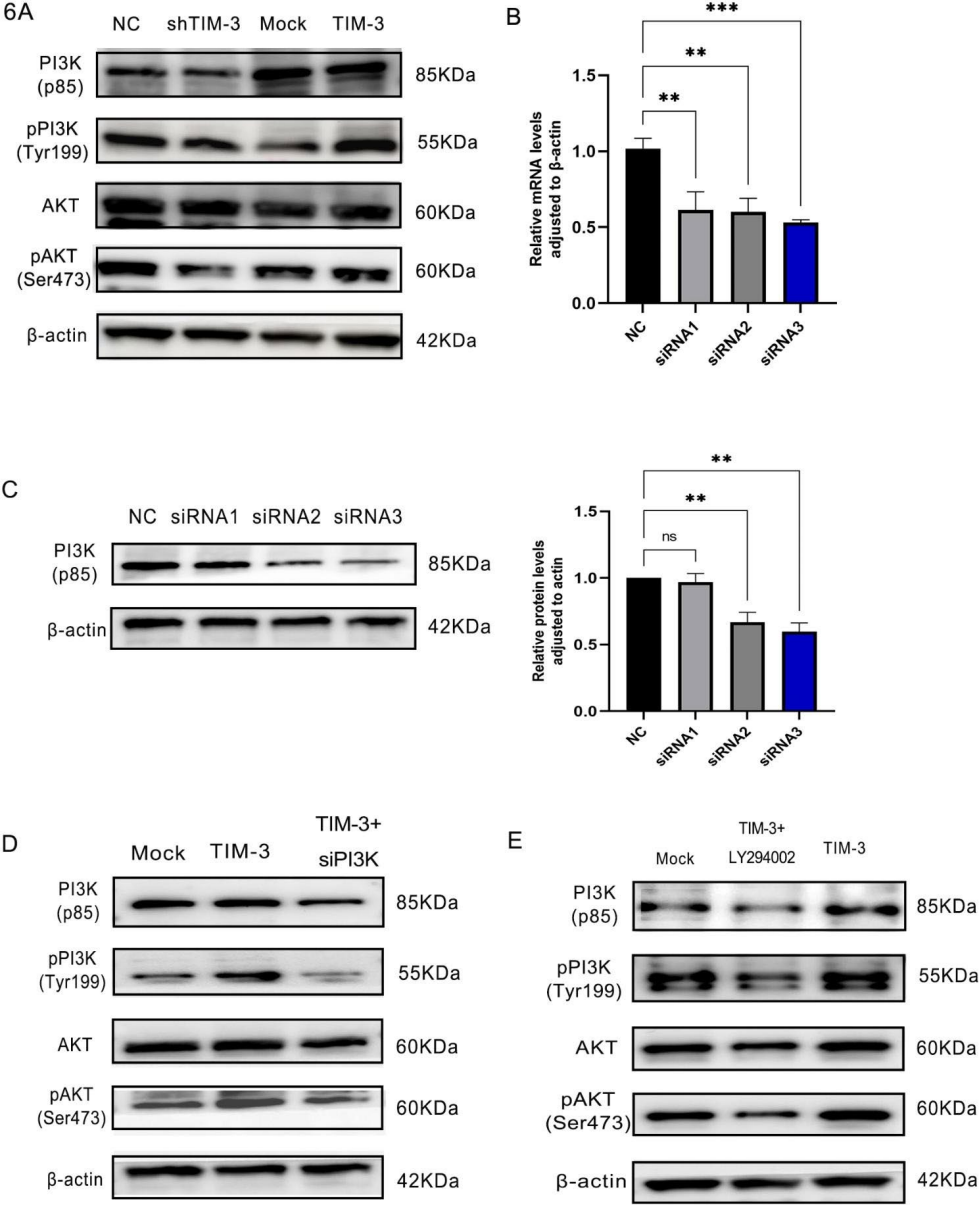
Rescue experiments. (**A**) Downregulation and upregulation of TIM-3 alter the expression of proliferation-related molecules through Western blotting. (**B**, **C**) Downregulation of PI3K was assessed by qRT-PCR and western blot analysis after transfection with three siRNAs. The first siRNA was selected for further investigation. (**D**) Downregulation of PI3K alters the expression of proliferation-related molecules through Western blotting. (**E**) LY294002 inhibits the expression of proliferation-related molecules through Western blotting. (*p < 0.05, **p < 0.01, ***p < 0.001, ****p < 0.0001)

To explore whether TIM-3 affects the PI3K and AKT signaling pathways central to cell proliferation, WB was used to assess activation of PI3K and AKT. Downregulation of TIM-3 decreased the expression of pAKT and pPI3K compared to that in the NC group (*p* < 0.01) (Fig. 6A).

PI3K knockdown and LY294002 inhibits the expression of PI3K/AKT pathway in Tim-3 overexpression ESCs.

To verify whether Tim-3 affects ESCs through PI3K/AKT pathway, three different siRNAs targeting PI3K and a negative control were synthesized by Obio Technology (Shanghai) Corp., Ltd, and the transfection rate was checked by q-PCR and WB, which was showed that the knockdown of TIM-3 expression by siRNA3 was effective (Fig. 6B and C). After transfecting the PI3K siRNA into the TIM-3 OE ESCs, WB was used to assess the expression of PI3K/AKT pathway, which showed that knocking down PI3K can downregulated the activity of TIM-3 through the PI3K/AKT signaling pathway (Fig. 6D).

TIM-3 NC and TIM-3 OE were incubated with the PI3K/AKT inhibitor LY294002 at different doses for 48 h. After adding 10 µL of CCK8 and incubating for one hour, the optical density (OD) was measured at 450 nm. These results indicated that the IC_50_ was 10.42 µM. TIM-3 NC and TIM-3 OE cells were treated with 10.42 µM LY294002, and the cellular proteins were extracted after 48 h and assayed by WB. To explore the effects of LY‑294,002 on the PI3K/AKT pathway in ESC, the expression levels of PI3K/AKT and p-PI3K/p-AKT were measured. As shown in Fig. 6E, the inhibitory effect of p-PI3K/p-AKT was evident, indicating that LY294002 downregulated the activity of TIM-3 through the PI3K/AKT signaling pathway.

### LY294002 inhibits the growth of endometriotic nodules in vivo

**Fig. 7:**
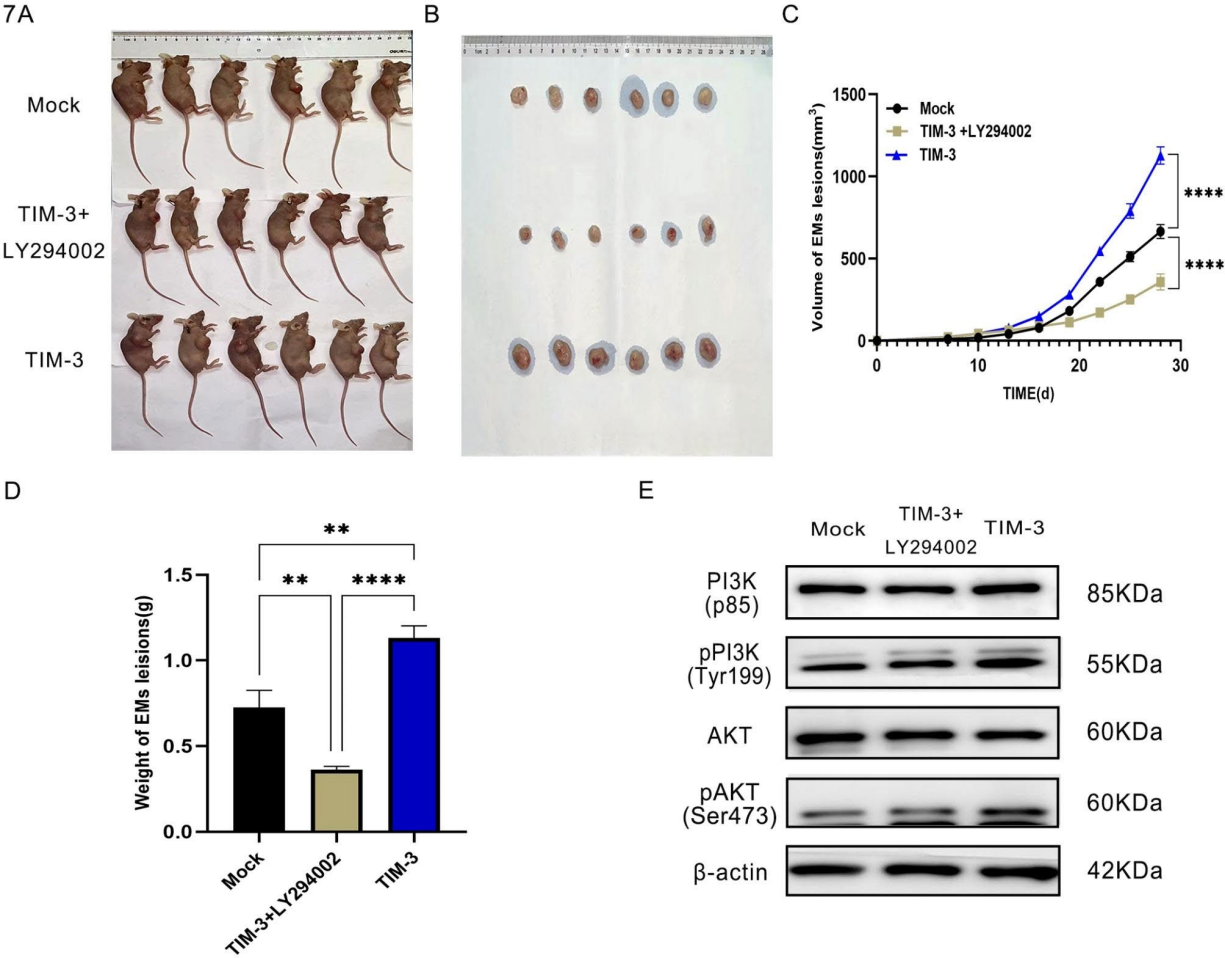
LY294002 downregulated the activity of TIM-3 through the PI3K/AKT signaling pathway in vivo. (**A**, **B**) LY294002 inhibits the proliferation of ESCs and endometriotic nodules in vivo. (**C**, **D**) LY294002 inhibits the growth of endometriotic nodules in vivo. (**E**) LY294002 inhibits the expression of proliferation-related molecules through Western blotting. (*p < 0.05, **p < 0.01, ***p < 0.001, ****p < 0.0001)

After establishing an endometriosis model in nude mice, the effect of LY294002 on the growth of the endometriotic nodules in vivo was assayed. First, TIM-3 NC and TIM-3 OE were administered by sub axillary injection into BALB/c nude mice, and one week after the injection, LY294002 (50 mg/kg) was administered intraperitoneally every four days. On the 25th day after modeling, the nude mice were euthanized and samples were harvested (Fig. 7A and B). The size and weight of the lesions are shown in Fig. 7C and D. The results of WB further verified that LY294002 inhibits the activity of TIM-3 through the PI3K/AKT signaling pathway (Fig. 7E).

### Analysis results of quality control and KEGG pathways

**Fig. 8:**
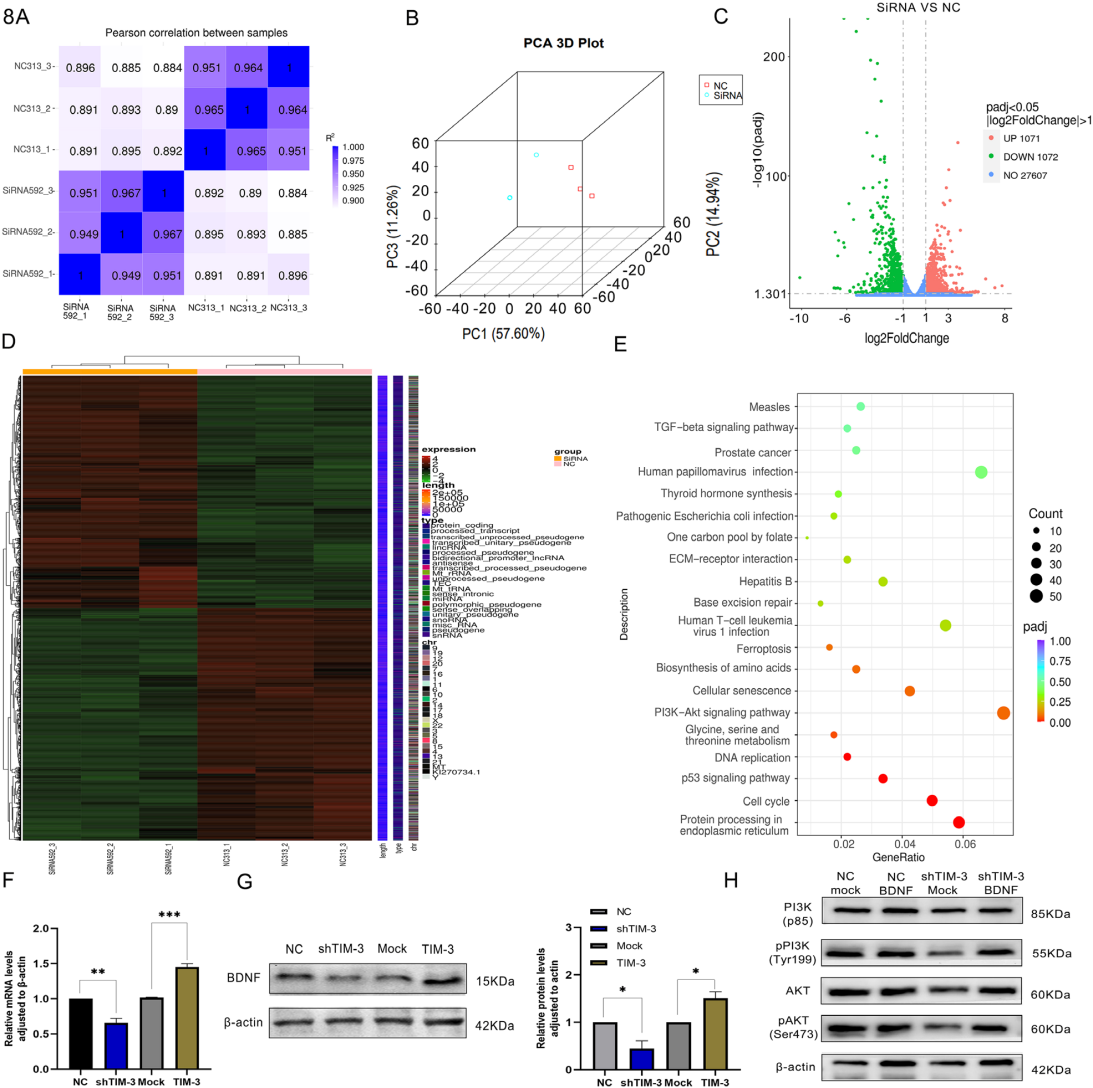
BDNF was sifted by RNA seq and verified by qRT‑PCR and WB. (**A**, **B**) The correlation of gene expression level between samples. (**C**) A volcano map of up-regulated or down-regulated genes. (**D**) 1071 genes were up-regulated and 1072 genes were down-regulated. (**E**) The top 20 enrichment of KEGG pathway. (**F**, **G**) BDNF was sifted and verified by qRT‑PCR and WB. (**H**) The expression of PI3K/AKT pathway molecules after BDNF overexpression plasmid to shTIM-3 ESCs

The correlation of gene expression level between samples is an important index to test the reliability of the experiment and whether the sample selection is reasonable. The closer the correlation coefficient is to 1, the higher the similarity of expression patterns between samples (Fig. 8A). The average number of readings per sample was about 45.87 million; the Q20 of all samples ranged from 96.89 to 97.3, Q30 ranged from 91.86 to 92.49%, and 90.19–94% of the samples could be mapped to the reference genome indicating that the sequencing results were reliable (Table 1). Figure 8B shows the correlation of gene expression levels among samples. The representative distribution of up-regulated or down-regulated genes in a volcano map is shown in Fig. 8C. Our data show that 1071 genes were up-regulated and 1072 genes were down-regulated according to the standard: DESeq2 *padj* < = 0.05, |log2FoldChange|>=1.0 (Fig. 8D). And the detail RNA seq analysis was in Fig S2.

**Table 1:** Analysis Results of Quality Control

sample	library	raw_ reads	Raw bases	Clean reads	clean_ bases	Error rate (%)	Q20 (%)	Q30 (%)	GC pct (%)	Reads ratio (%)
SiRNA_1	FRAS220067465-1r	44,864,676	6.73G	40,664,156	6.1G	0.03	96.89	91.86	51.91	90.63736
SiRNA_2	FRAS220067466-1r	47,935,430	7.19G	43,729,822	6.56G	0.03	97.3	92.49	50.56	91.22651
SiRNA_3	FRAS220067467-1r	45,061,496	6.76G	40,766,746	6.12G	0.03	97.25	92.48	50.74	90.46914
NC_1	FRAS220067468-1r	46,394,254	6.96G	41,842,372	6.28G	0.03	97.23	92.39	51.13	90.18869
NC_2	FRAS220067469-1r	45,726,546	6.86G	42,948,358	6.44G	0.03	97.2	92.36	51.2	93.92434
NC_3	FRAS220067470-1r	45,258,206	6.79G	42,545,084	6.38G	0.03	97.06	91.96	51.84	94.00524

Cluster Profiler software was used to analyze the enrichment of KEGG pathway in different gene sets, and *padj* < 0.05 was considered as the threshold of significant enrichment. Figure 8E displayed the top 20 enrichments of KEGG pathways, while a more detailed representation of the KEGG pathway enrichment could be found in Fig S3. Through the results of CCK8 and EDU assays, we chose the proliferation related PI3K/AKT pathway to do the further study. We found TIM-3 regulates the proliferation by BDNF-mediated PI3K/AKT pathway, which was verified by qRT‑PCR and WB (Fig. 8F and G). To further verified the function of BDNF, we transfected BDNF overexpression plasmid to shTIM-3 ESCs, and used WB to test the expression of PI3K/AKT pathway molecules (Fig. 8H).

### TIM-3 affects the proliferation of endometriotic foci by BDNF-mediated PI3K/AKT pathway in vivo

**Fig. 9:**
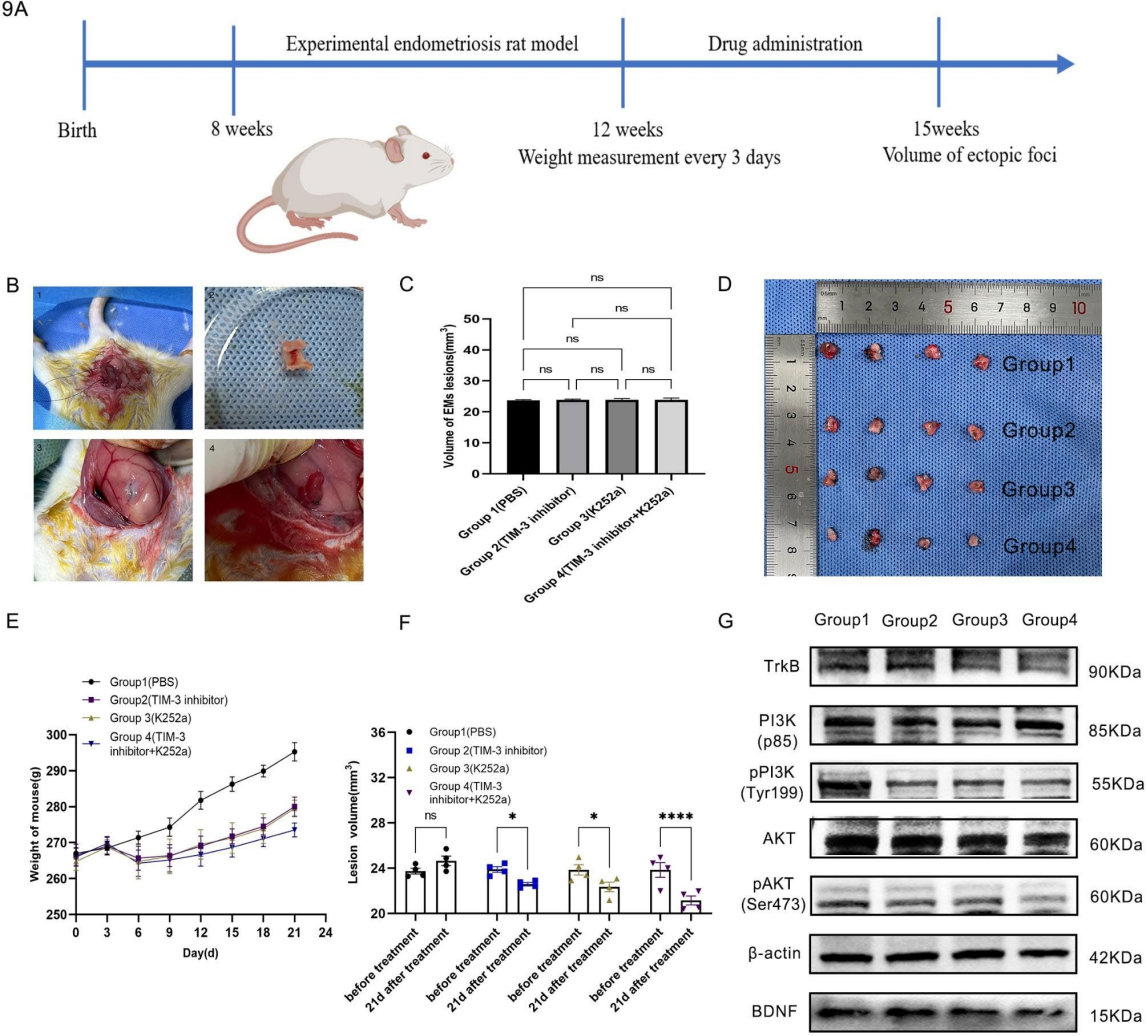
TIM-3 affects the proliferation of endometriotic foci by BDNF-mediated PI3K/AKT pathway in vivo. (**A**) Flow diagram of experiment arrangement. (**B**) The modeling pictures. (**C**) The volume of foci before treatments. (**D**, **E**) The volume of foci after treatments. (**F**) The weight change of rats in the period of treatments. (**G**) The expression of PI3K/AKT related molecules. (*p < 0.05, **p < 0.01, ***p < 0.001, ****p < 0.0001)

After 4 weeks following the first operation, we performed a second laparotomy to determine the success rate of modeling and measure the volumes of foci(length*width*height). And 16 rats with the successful model were randomly divided into four groups and the volume of the foci had no significant difference(*p* > 0.05) (Fig. 9B, C). The weight of rats and volume of foci were measured after all rats were sacrificed with Aver din anesthesia (Fig. 9D, E, F). The foci were immediately placed in an ultra-low temperature freezer for subsequent WB experiments. The expression pPI3K and pAKT was extremely inhibited in group4(Fig. 9G).

## Discussion

Reflux of menstrual blood in endometriosis is only the cause of endometriosis, while the key to the pathogenesis is the eutopic endometrium itself. The change in eutopic endometrial stem/progenitor cells or their microenvironment is likely the root cause. Testing of tissue samples and cells showed that TIM-3 was highly expressed in Ec and Eu of ovarian endometriosis. Knockdown of TIM-3 in ESCs decreased the proliferation and clone formation abilities, suggesting that TIM-3 has a key effect on the proliferation of endometriosis cells. The current results provide new perspectives on the role of TIM-3 in endometriosis.

Recent research has shown that TIM-3 expression is correlated with autoimmune diseases, cancer, and chronic infectious illnesses. TIM-3/Gal-9 signaling has a negative regulatory effect on rheumatoid arthritis (RA). Furthermore, Gal-9 inhibits RA disease progression through regulatory T cells (Lee et al. 2011a, b). The expression level of TIM-3 on T cells of the cerebrospinal fluid clones from multiple sclerosis (MS) patients was reported to be higher than that in the control group (Yang et al. 2008). Some researchers have found that the level of TIM-3 is increased in hepatic cellular cancer (HCC) and that it is negatively related to the survival time of patients. Inhibition of the TIM-3/Gal-9 signaling pathway can, therefore, enhance the effect of T cells and restrain T cell depletion (Li et al. 2012; Zhao et al. 2020; Ngiow et al. 2011). TIM-3 can exert a negative immunomodulatory effect by inhibiting the function of NK cells during chronic HIV infection (Finney et al. 2013; Klibi et al. 2009). TIM-3 is expressed by NK cells in patients with acute myelocytic leukemia (AML) and it can enhance cytotoxicity related to patient prognosis. Blocking both PD-1/PD-L1 and TIM-3/Gal-9 signaling pathways can inhibit T cell exhaustion in patients with hematological malignant disease (Rakova et al. 2021; Tan et al. 2020). NSCLC patients with TIM-3^+^Treg expression have poor clinical features and more advanced disease (Gao et al. 2012). A meta-analysis that included 869 Asian patients in seven different studies of the relationship between TIM-3 expression and the prognosis of urothelial, renal cell cancer, non-small-cell lung cancer, hepatoma, colorectal carcinoma, gastric cancer, and cervical carcinoma found that TIM-3 expression was negatively related to overall survival and tumor stage (Zhang et al. 2017). However, the expression of TIM-3 in ESCs and endometriotic lesions and its role in the progression of endometriosis are not clear. By testing human tissue samples, we observed that the expression of TIM-3 in Ec and Eu was higher than that in normal tissues at the mRNA and protein levels, suggesting that TIM-3 is linked to the pathogenesis and progression of endometriosis. This study indicates that TIM-3, which has a direct influence on the proliferation of endometriosis cells, is linked to the pathogenesis and progression of endometriosis lesions and may become a therapeutic target for endometriosis. Previous studies have similarly found that TIM-3 is overexpressed in esophageal squamous cell carcinoma tissues (Shan et al. 2016).

Similar to malignant tumors, one of the characteristics of endometriosis is abnormal cell proliferation. Alterations in cell proliferation, clone formation, and endometriosis focus-forming ability were verified in ESCs and animal experiments to explore whether TIM-3 affects the growth of endometriosis lesions. The results show that low expression of TIM-3 inhibited proliferation and clone formation in ESCs, as well as the growth of endometriosis foci in nude mice. Additionally, the results of the apoptosis assay indicate that TIM-3 overexpression had an antiapoptotic effect.

A number of studies have also shown that the downregulation of TIM-3 is closely related to metastasis and poor prognosis in prostate and colon cancer (Wu et al. 2017; Sun et al. 2017) and inhibits the proliferation and invasion of renal clear cell carcinoma and HeLa cells (Yuan et al. 2014; Cao et al. 2016). However, unlike the role in tumors, the results of wound healing, Transwell® migration, and invasion experiments in vitro showed that overexpression/low expression of TIM-3 only affected the migration of ESCs.

A multitude of studies have clearly shown that the PI3K/AKT signaling pathway plays a central role in cell growth, cell cycle progression, immunoreaction, and apoptosis (Li et al. 2017). Therefore, we inferred that the PI3K/AKT signaling pathway affects cell growth through cell phenotype experiments. Using WB blotting, we assayed for changes in PI3K/pPI3K and AKT/pAKT expression, and the results showed that low TIM-3 expression inhibited proliferation through the PI3K/AKT signaling pathway. Knocking down PI3K and LY294002 were used as PI3K/AKT pathway inhibitors to verify the mechanism of TIM-3 function in vitro and in vivo. LY294002 was used as a PI3K/AKT pathway inhibitor to verify the mechanism of TIM-3 function in vitro and in vivo. LY294002 significantly inhibited the growth of ESCs in a dose-dependent manner in the CCK8 assay. The results of our WB assays and nude mouse experiments show that PI3K knock down and LY294002 downregulated the activity of TIM-3 through the PI3K/AKT signaling pathway.

BDNF is a member of a unique family of polypeptide growth factors, neurotrophins, which influence the proliferation, differentiation, survival, and death of neuronal and non-neuronal cells (Chao 2003; Lee et al. 2001). Several studies investigated the BDNF-mediated PI3K/Akt signaling pathway as a therapeutic method (Long et al. 2016; Li et al. 2020; Song et al. 2017; Bao et al. 2014; Desmet and Peeper 2006). Through results of RNA seq, BDNF was sifted and verified by qRT-PCR, and WB. In order to further explore the effects of BDNF in the proliferation of ESCs and endometriosis, we transfected BDNF overexpression plasmid to shTim-3 ESCs and gave Tim-3 inhibitor and K252a to the rats’ endometriosis model. The results showed that Tim-3 regulated the proliferation by BDNF-mediated PI3K/Akt signaling pathway in vitro and vivo.

Excessive proliferation and migration of ESCs have been identified as pivotal factors contributing to the progression of endometriosis, as established in previous studies (Chen et al. 2018; Fu et al. 2021). Research has consistently substantiated that TIM-3 serves as a facilitator of cell proliferation and migration in various cancers. Notably, TIM-3 knockout resulted in a marked inhibition of cell proliferation. By investigating proliferation-associated pathways both in vivo and in vitro, our study introduces the novel concept that TIM-3 may exert its influence on ESCs proliferation and endometriotic lesion development through the BDNF mediated PI3K/AKT pathway. This discovery further corroborates the potential role of TIM-3 as a regulator in the pathogenesis of endometriosis. These findings offer valuable insights into the underlying mechanisms of endometriosis and present a promising new therapeutic target for clinical management of this condition. However, our study has some limitations. Firstly, the study was performed with eutopic ESC only. Due to limited experimental techniques, the use of ectopic ESC for a series of in-vitro cell experiments had to be abandoned, because it was not possible to obtain high-purity ectopic ESC. Secondly, it is regrettable that the study only made a preliminary exploration of the possible molecular mechanisms and did not further expose whether TIM-3 has a directed relationship with BDNF. The Future study should delve deeper into the molecular mechanisms of TIM-3 in endometriosis.

## Conclusion

**Fig. 10:**
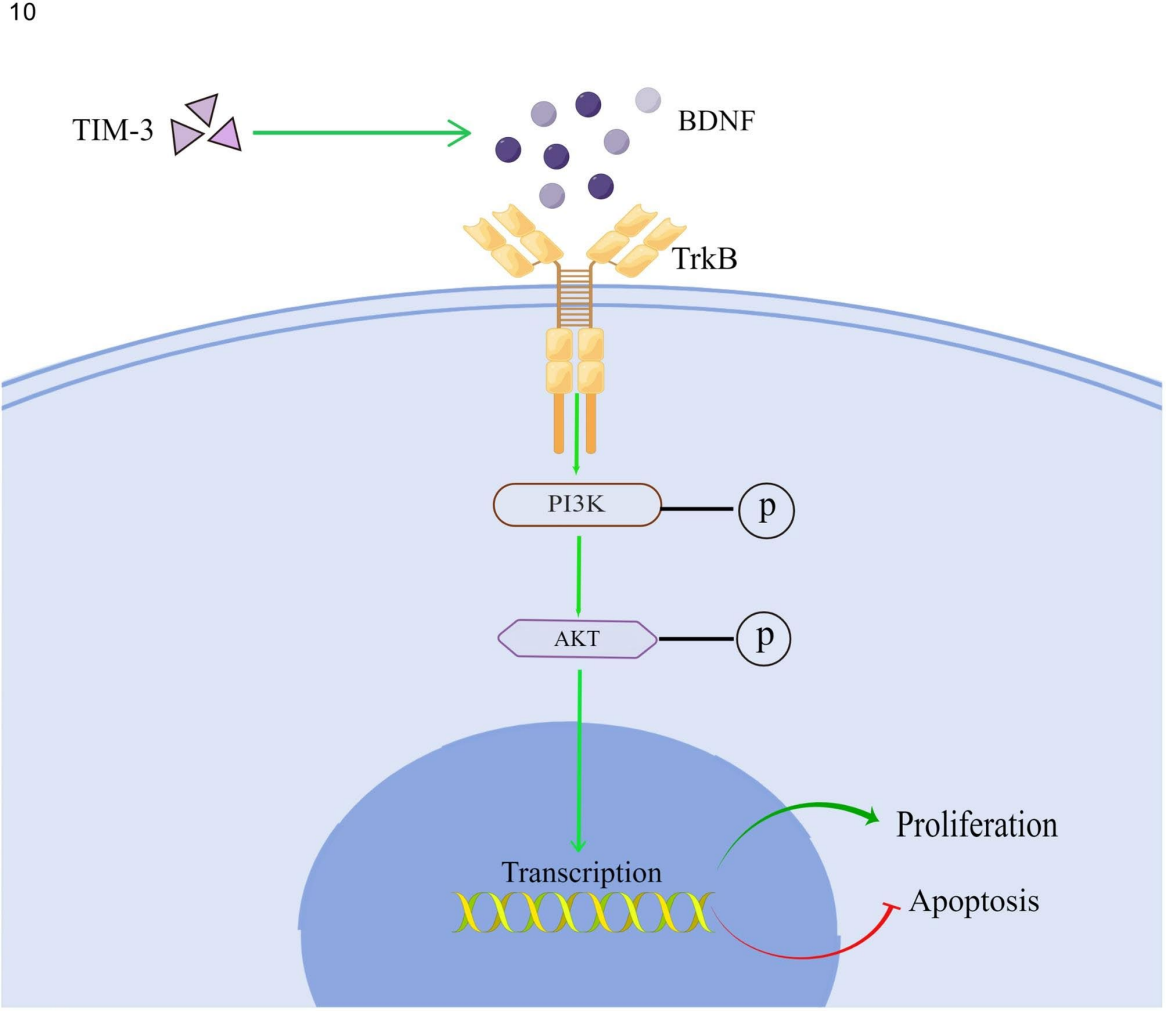
TIM-3 promote the proliferation by BDNF–mediated PI3K/AKT axis

In summary, our study shows that TIM-3 is involved in the proliferation of endometriosis cells by BDNF-mediated PI3K/AKT axis (Fig. 10). Our research will provide a new perspective for the pathogenesis and treatment of endometriosis.

### Electronic supplementary material

Below is the link to the electronic supplementary material.

Supplementary Material 1

Supplementary Material 2

Supplementary Material 3

## Data Availability

The datasets used during the current study are available from the corresponding author on reasonable request.

## Abbreviations

TIM-3: T cell immunoglobulin and mucin domain-containing molecule-3
CCK8: Cell Counting Kit-8
EDU: 5-ethynyl-2’-deoxyuridine
BDNF: Brain-derived neurotrophic factor
PI3K/AKT: Phosphatidylinositol 3 kinase/protein kinase B
EMs: Endometriosis
HAVCR2: Hepatitis A virus cellular receptor 2
ESCs: Endometrial stromal cells
Ec: Ectopic endometrium tissues
Eu: Eutopic endometrium tissues
NE: Normal endometrium tissues
IHC: Immunohistochemistry
hESC: Human endometrial stromal cell line
shRNAs: Short hairpin RNAs
MOI: Multiplicity of infection
qRT‑PCR: Quantitative Real‑Time Polymerase Chain Reaction
WB: Western blotting
RIPA: Radioimmunoprecipitation
SDS-PAGE: Sulfate-polyacrylamide gel electrophoresis
HRP: Horseradish peroxidase
OD: Optical density
FITC: Fluorescein isothiocyanate
PI: Propidium Iodide
QC: Quality control
siRNA: Small interfering RNA
