# Supplementary figures and images for: TIM-3 regulates the proliferation by BDNF-mediated PI3K/AKT axis in the process of endometriosis
Source: Mol Med. 2023 Dec 19;29:170. doi: 10.1186/s10020-023-00768-6 (PMC10731854; doi:10.1186/s10020-023-00768-6)

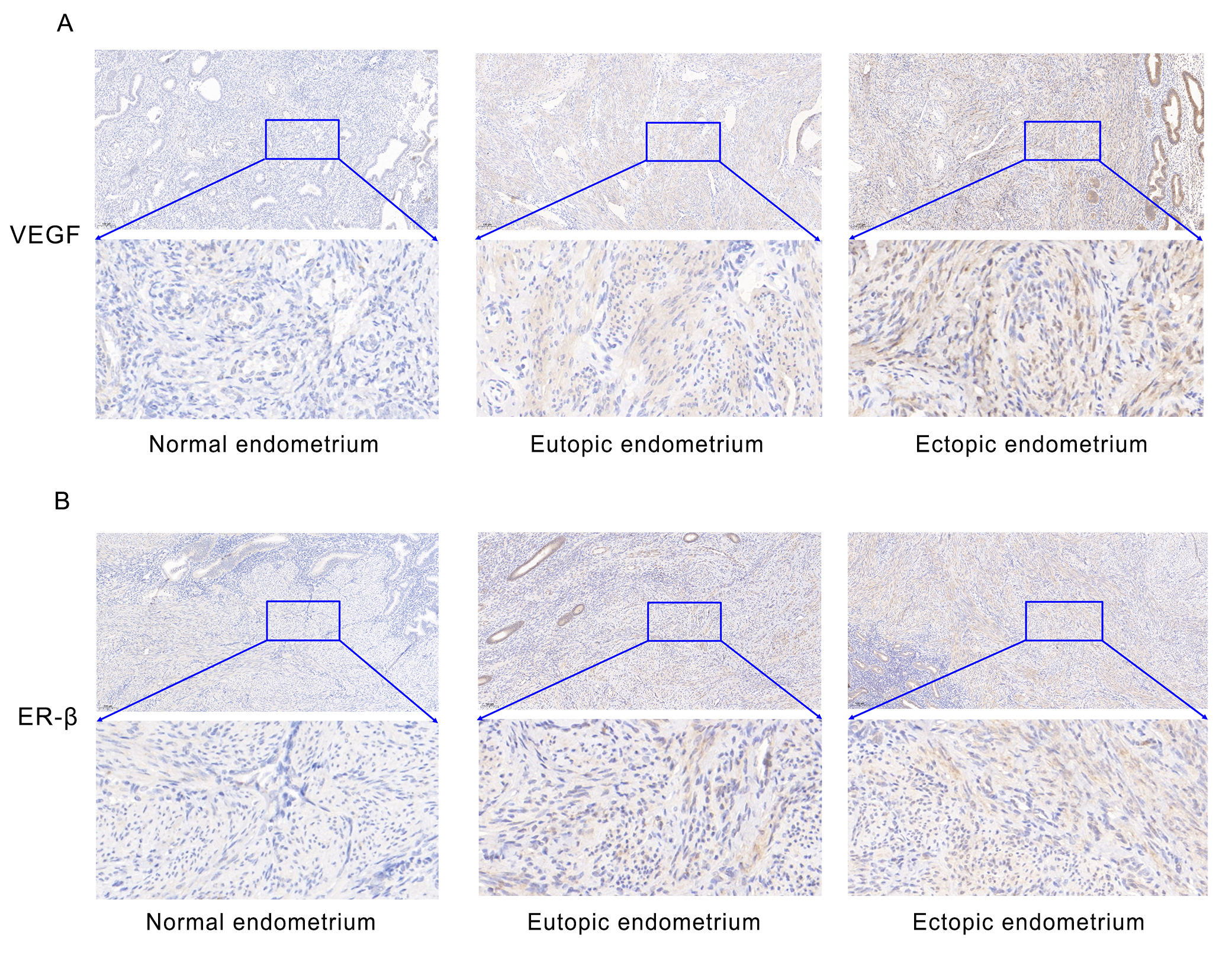

Supplement: Supplementary file 1 — Supplementary Material 1 [file 10020_2023_768_MOESM1_ESM.jpg]
